# Supplementary material for: Dexmedetomidine use in pediatric strabismus surgery: A systematic review and meta-analysis
Source: PLoS One. 2020 Oct 12;15(10):e0240553. doi: 10.1371/journal.pone.0240553 (PMC7549777; doi:10.1371/journal.pone.0240553)
Supplement: S1 Table — (DOCX) [file pone.0240553.s002.docx]

**Table S1. Search Strategy**

| Recent queries in PubMed on April 4, 2020 | | |
| --- | --- | --- |
| Search | Query | Items found |
| #1 | Search "strabismus"[MeSH Terms] | 15846 |
| #2 | Search "strabismus"[All Fields] | 21793 |
| #3 | Search "ophthalmologic surgical procedures"[MeSH Terms] | 109328 |
| #4 | Search "ophthalmologic surgical procedures"[All Fields] | 12100 |
| #5 | Search ("ophthalmologic"[All Fields] AND "surgical"[All Fields] AND "procedures"[All Fields]) | 12330 |
| #6 | Search ("eye"[All Fields] AND "surgery"[All Fields]) OR "eye surgery"[All Fields] | 87117 |
| #7 | Search "ophthalmic"[All Fields] AND "surgery"[All Fields] | 21556 |
| #8 | Search "ophthalmic surgery"[All Fields] | 4451 |
| #9 | Search "dexmedetomidine"[MeSH Terms] | 3478 |
| #10 | Search "dexmedetomidine"[All Fields] | 5848 |
| #11 | Search "precedex"[All Fields] | 32 |
| #12 | Search ((((((("strabismus"[MeSH Terms]) OR "strabismus"[All Fields]) OR "ophthalmologic surgical procedures"[MeSH Terms]) OR "ophthalmologic surgical procedures"[All Fields]) OR (("eye"[All Fields] AND "surgery"[All Fields]) OR "eye surgery"[All Fields])) OR (("ophthalmologic"[All Fields] AND "surgical"[All Fields] AND "procedures"[All Fields]))) OR ("ophthalmic"[All Fields] AND "surgery"[All Fields])) OR "ophthalmic surgery"[All Fields] | 179691 |
| #13 | Search (("precedex"[All Fields]) OR "dexmedetomidine"[All Fields]) OR "dexmedetomidine"[MeSH Terms] | 5850 |
| #14 | Search (((("precedex"[All Fields]) OR "dexmedetomidine"[All Fields]) OR "dexmedetomidine"[MeSH Terms])) AND (((((((("strabismus"[MeSH Terms]) OR "strabismus"[All Fields]) OR "ophthalmologic surgical procedures"[MeSH Terms]) OR "ophthalmologic surgical procedures"[All Fields]) OR (("eye"[All Fields] AND "surgery"[All Fields]) OR "eye surgery"[All Fields])) OR (("ophthalmologic"[All Fields] AND "surgical"[All Fields] AND "procedures"[All Fields]))) OR ("ophthalmic"[All Fields] AND "surgery"[All Fields])) OR "ophthalmic surgery"[All Fields]) | 111 |
| Recent queries in EMBASE on April 4, 2020 | | |
| #1 | 'dexmedetomidine'/exp OR 'dexmedetomidine' | 11166 |
| #2 | 'precedex'/exp OR precedex | 10890 |
| #3 | 'strabismus'/exp OR strabismus | 35641 |
| #4 | ophthalm* AND surgical AND procedure* | 16522 |
| #5 | ophthalmic AND ('surgery'/exp OR surgery) | 40866 |
| #6 | #1 OR #2 | 11166 |
| #7 | #3 OR #4 OR #5 | 86547 |
| #8 | #6 AND #7 | 80 |
| Recent queries in Cochrane database on April 04, 2020 | | |
| #1 | MeSH descriptor: [Strabismus] explode all trees | 510 |
| #2 | Strabismus | 1280 |
| #3 | MeSH descriptor: [Ophthalmologic Surgical Procedures] explode all trees | 5736 |
| #4 | Ophthalm* surgical procedure* | 1537 |
| #5 | Ophthalmic surgery | 2832 |
| #6 | MeSH descriptor: [Dexmedetomidine] explode all trees | 1632 |
| #7 | dexmedetomidine | 4681 |
| #8 | precedex | 68 |
| #9 | #1 or #2 or #3 or #4 or #5 | 8944 |
| #10 | #6 or #7 or #7 | 4681 |
| #11 | #9 AND #10 | 54 |
|  |  |  |
| Recent queries in Scopus on April 04, 2020 | | |
| #1 | TITLE-ABS-KEY (strabismus) | 29970 |
| #2 | TITLE-ABS-KEY (ophthalm* AND surgical AND procedure*) | 19186 |
| #3 | TITLE-ABS-KEY (ophthalmic AND surgery) | 14344 |
| #4 | TITLE-ABS-KEY (dexmedetomidine) | 9524 |
| #5 | TITLE-ABS-KEY (precedex) | 435 |
| #6 | (TITLE-ABS-KEY (strabismus)) OR (TITLE-ABS-KEY (ophthalm* AND surgical AND procedure*)) OR (TITLE-ABS-KEY (ophthalmic AND surgery)) | 56928 |
| #7 | (TITLE-ABS-KEY (dexmedetomidine)) OR (TITLE-ABS-KEY (precedex)) | 9526 |
| #8 | (TITLE-ABS-KEY (strabismus)) OR (TITLE-ABS-KEY (ophthalm* AND surgical AND procedure*)) OR (TITLE-ABS-KEY (ophthalmic AND surgery)) AND (TITLE-ABS-KEY (dexmedetomidine)) OR (TITLE-ABS-KEY (precedex)) | 53 |
| Recent queries in Web of Sciences on April 4, 2020 | | |
| #1 | TS=(Strabismus) | 8115 |
| #2 | TS=(dexmedetomidine) | 6345 |
| #3 | TS=(precedex) | 32 |
| #4 | TS=(ophthalm* AND surgical AND procedure*) | 2319 |
| #5 | TS=(ophthalmic AND ('surgery'/exp OR surgery)) | 4985 |
| #6 | #3 OR #2 | 6346 |
| #7 | #5 OR #4 | 6740 |
| #8 | #7 OR #1 | 14532 |
| #9 | #8 AND #6 | 45 |
