# Supplementary figures and images for: Dexmedetomidine use in pediatric strabismus surgery: A systematic review and meta-analysis
Source: PLoS One. 2020 Oct 12;15(10):e0240553. doi: 10.1371/journal.pone.0240553 (PMC7549777; doi:10.1371/journal.pone.0240553)

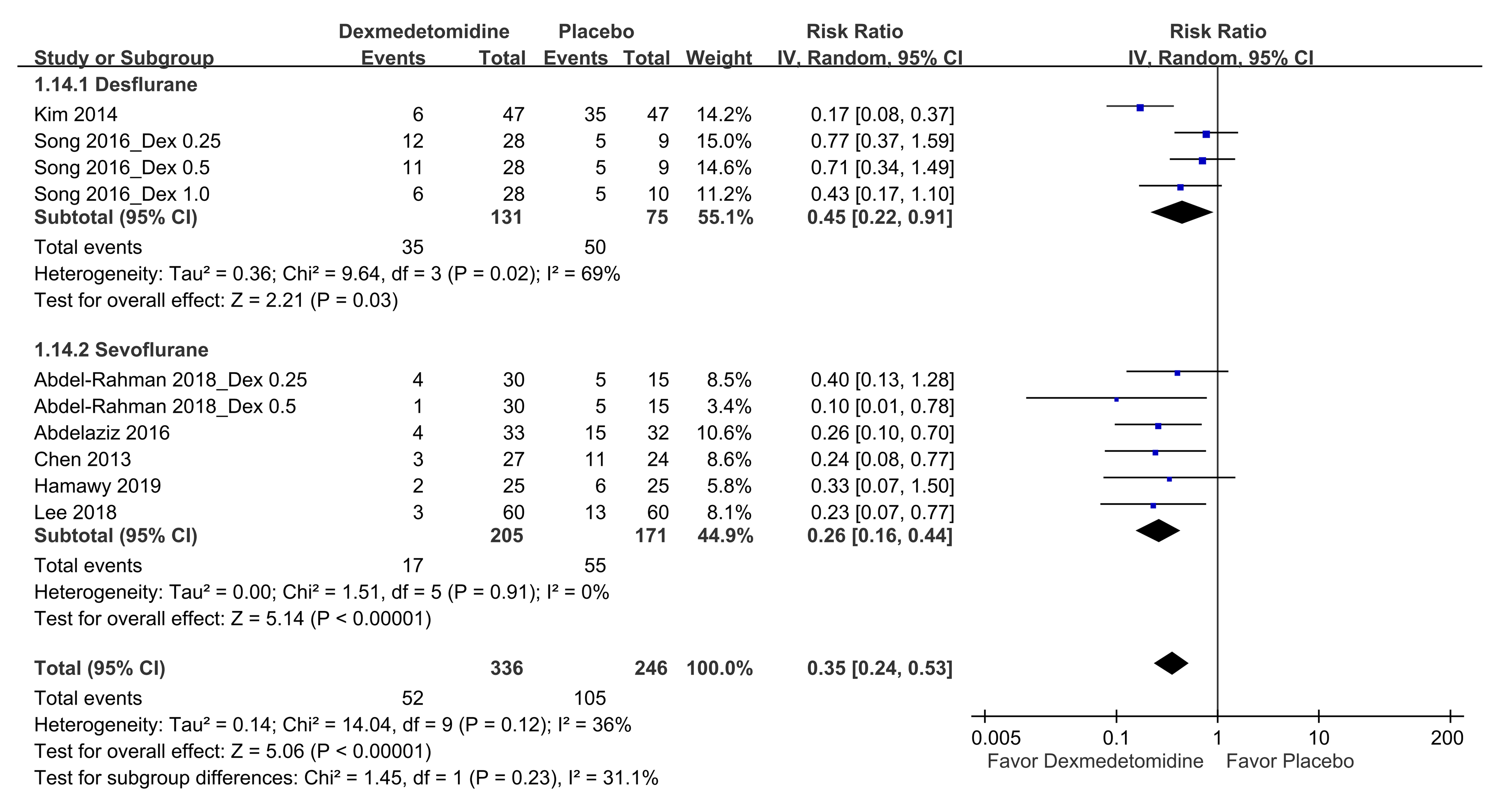

Supplement: S1 Fig — (TIF) [file pone.0240553.s003.tif]

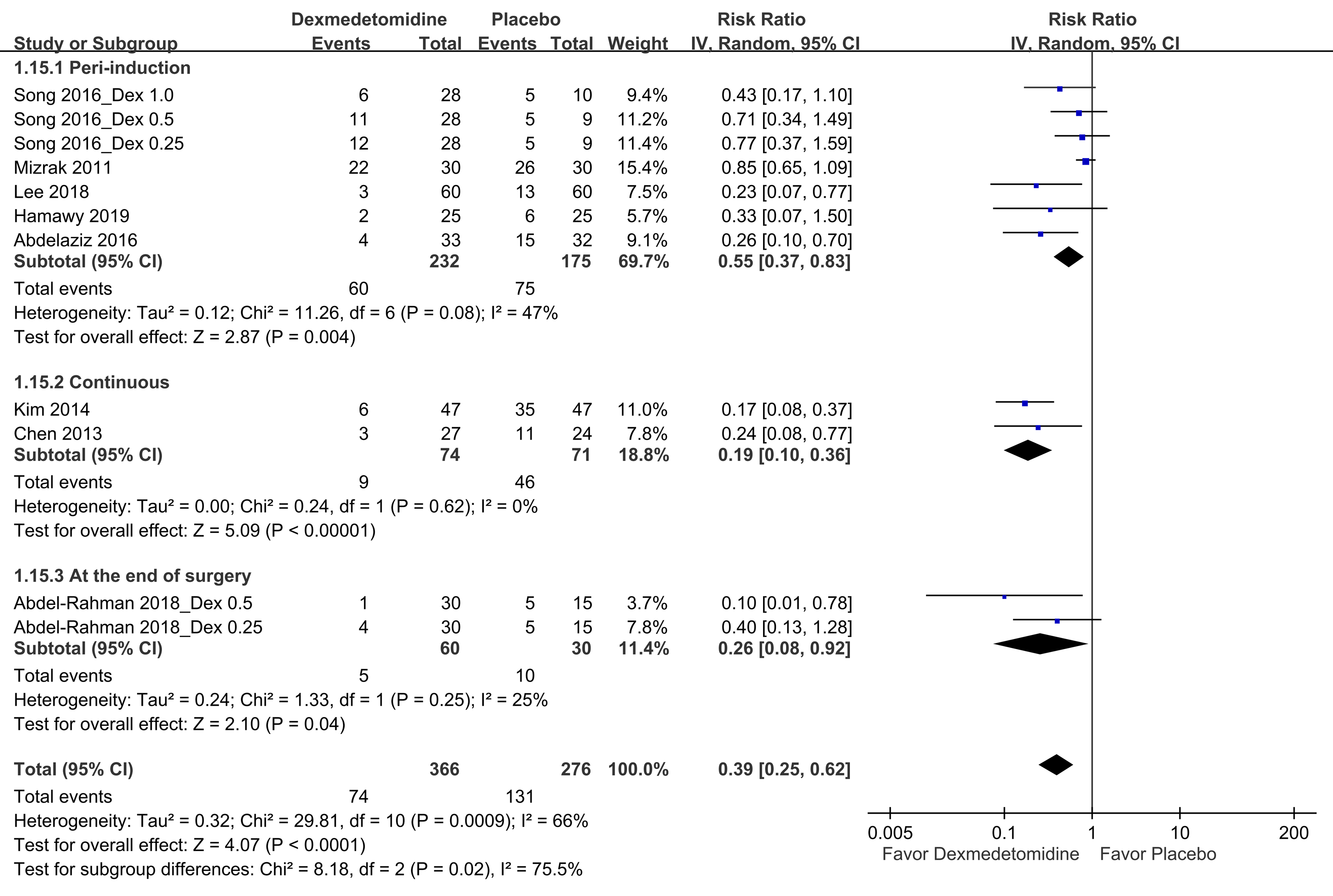

Supplement: S2 Fig — (TIF) [file pone.0240553.s004.tif]

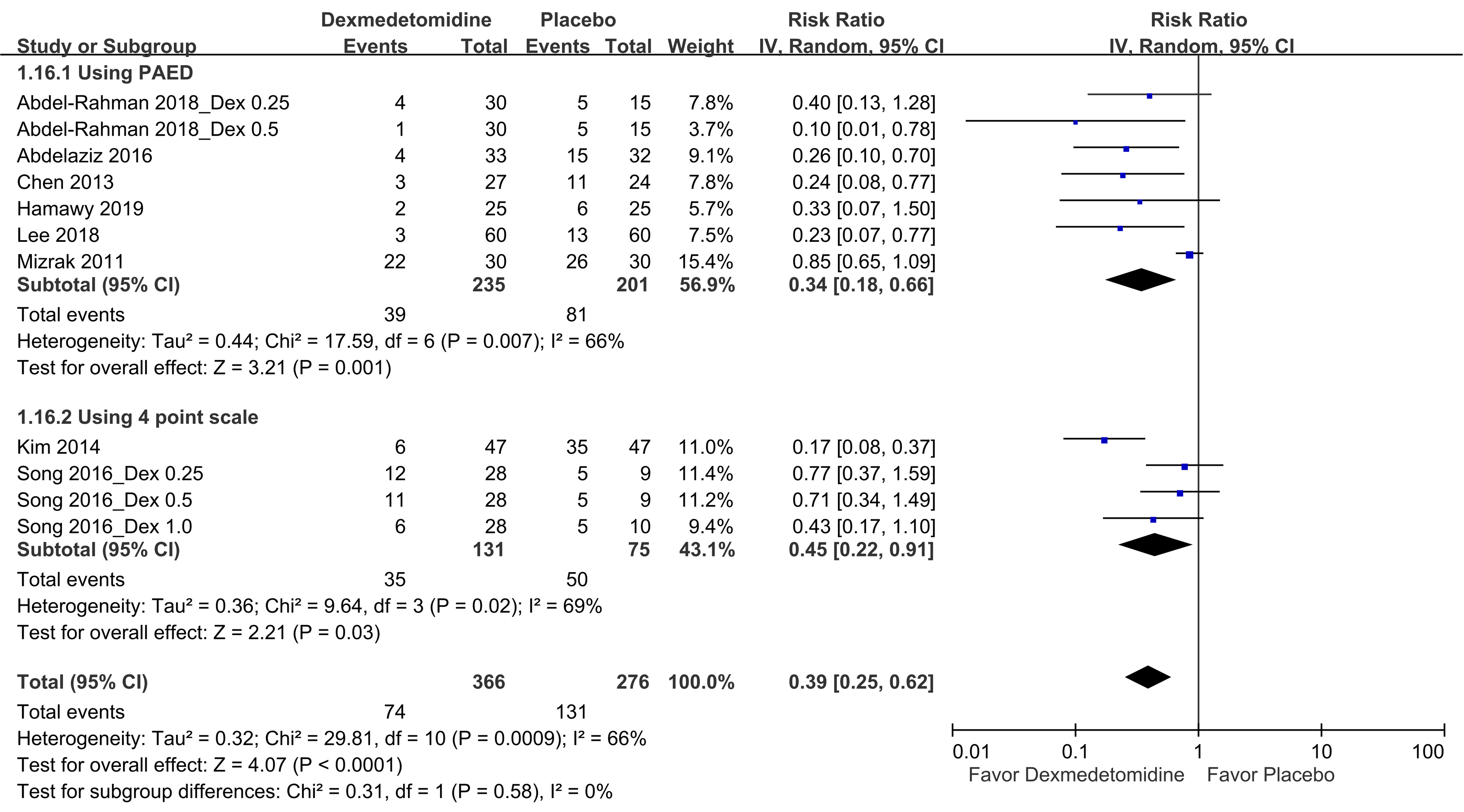

Supplement: S3 Fig — (TIF) [file pone.0240553.s005.tif]

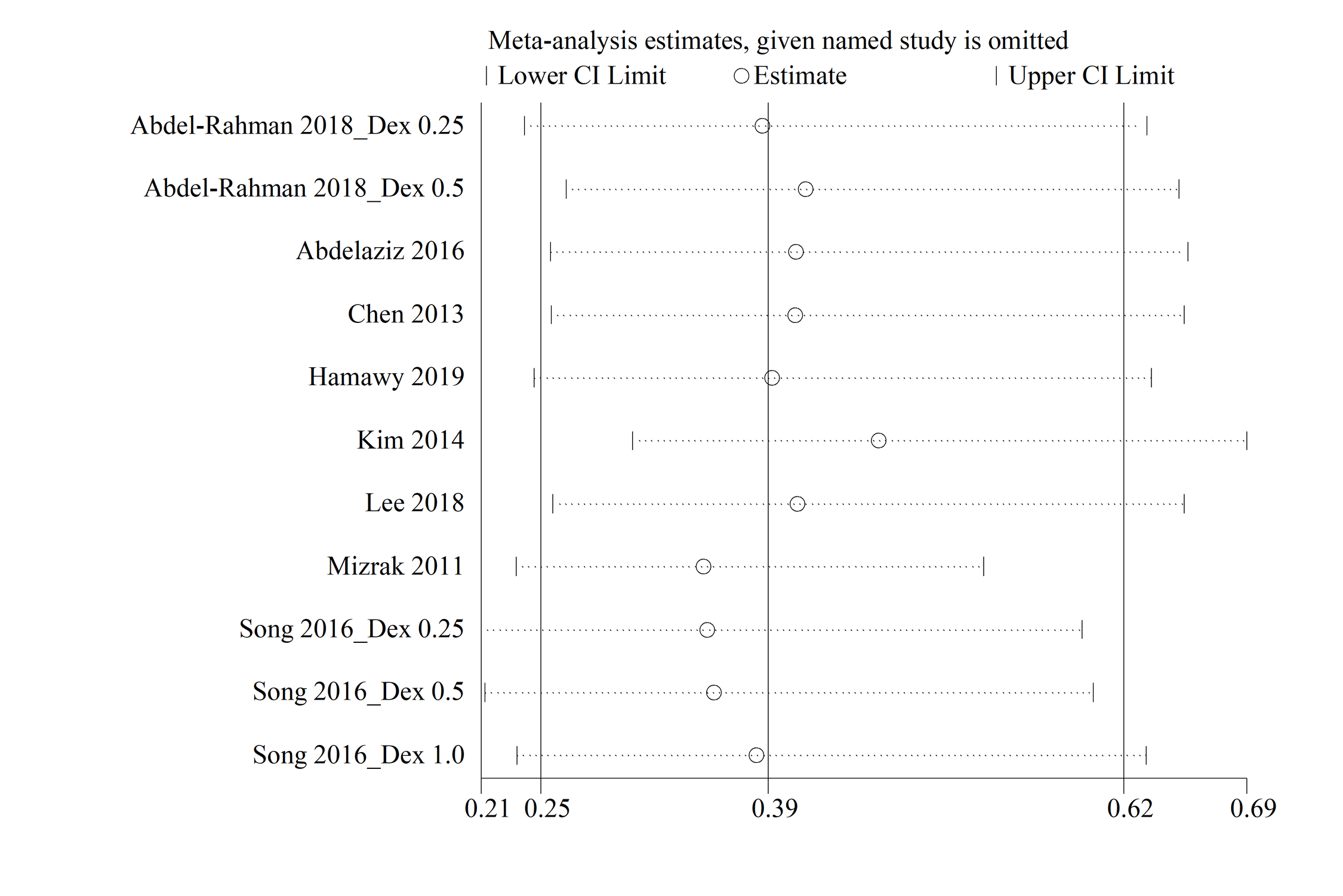

Supplement: S4 Fig — (TIF) [file pone.0240553.s006.tif]

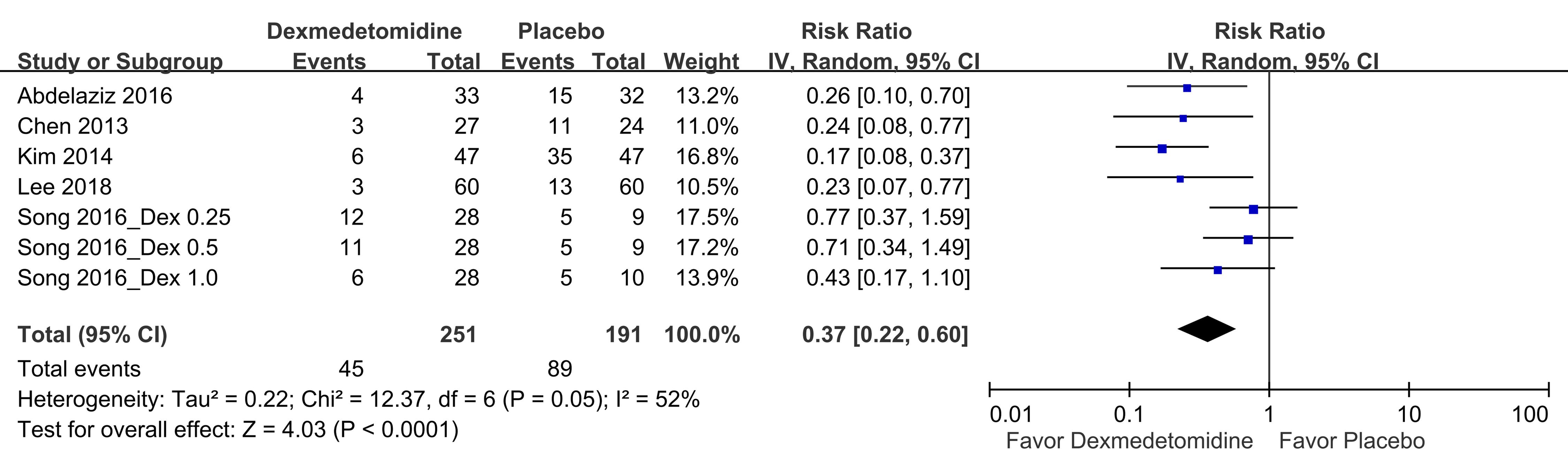

Supplement: S5 Fig — (TIF) [file pone.0240553.s007.tif]

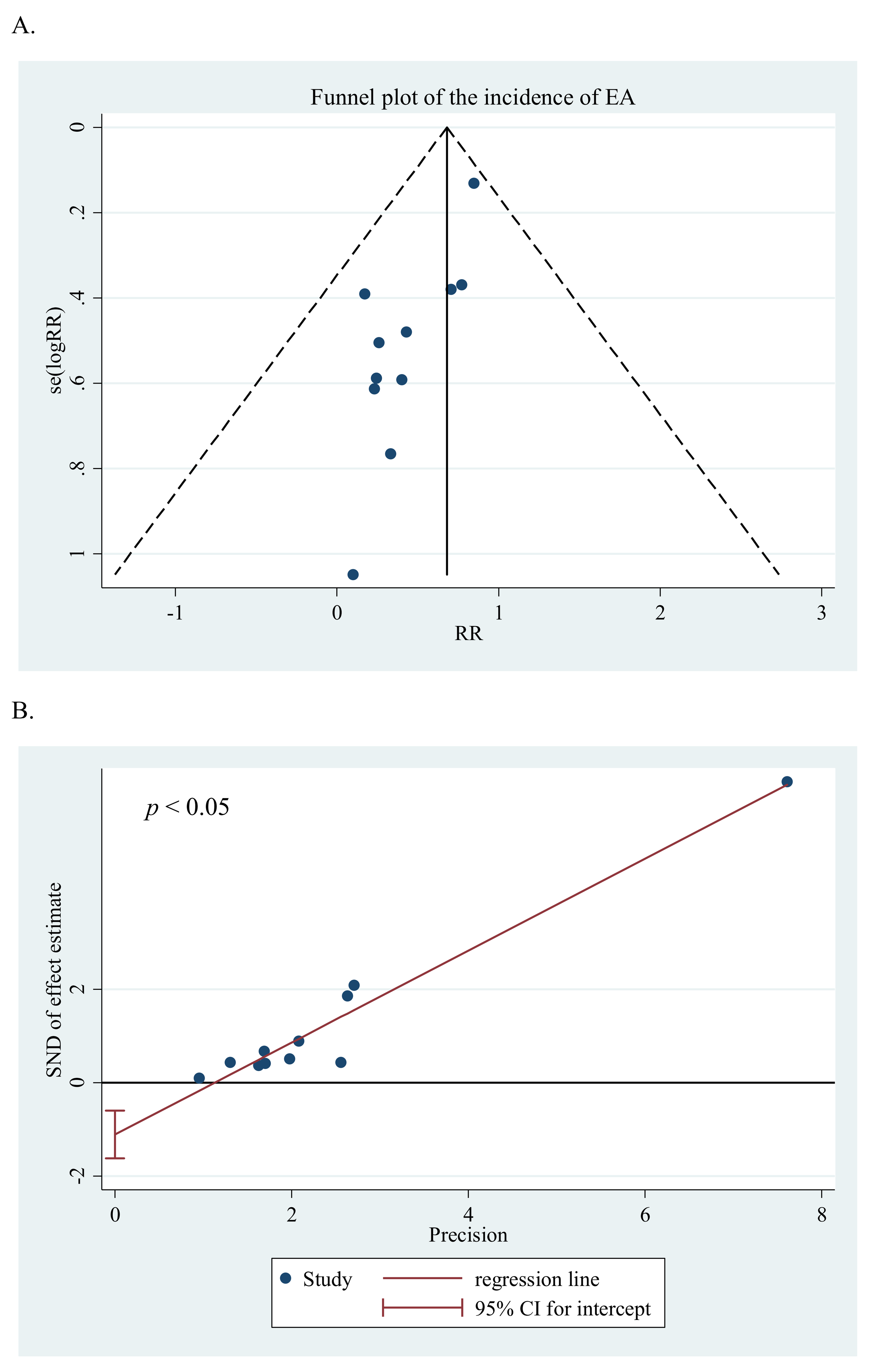

Supplement: S6 Fig — (A) Funnel plots and (B) Egger’s test results for EA incidence. The pseudo 95% CIs computed as part of the analyses were used to obtain the funnel plots and Egger’s test results. The pseudo 95% CIs corresponded to the expected 95% CIs for a given standard error (SE). (TIF) [file pone.0240553.s008.tif]

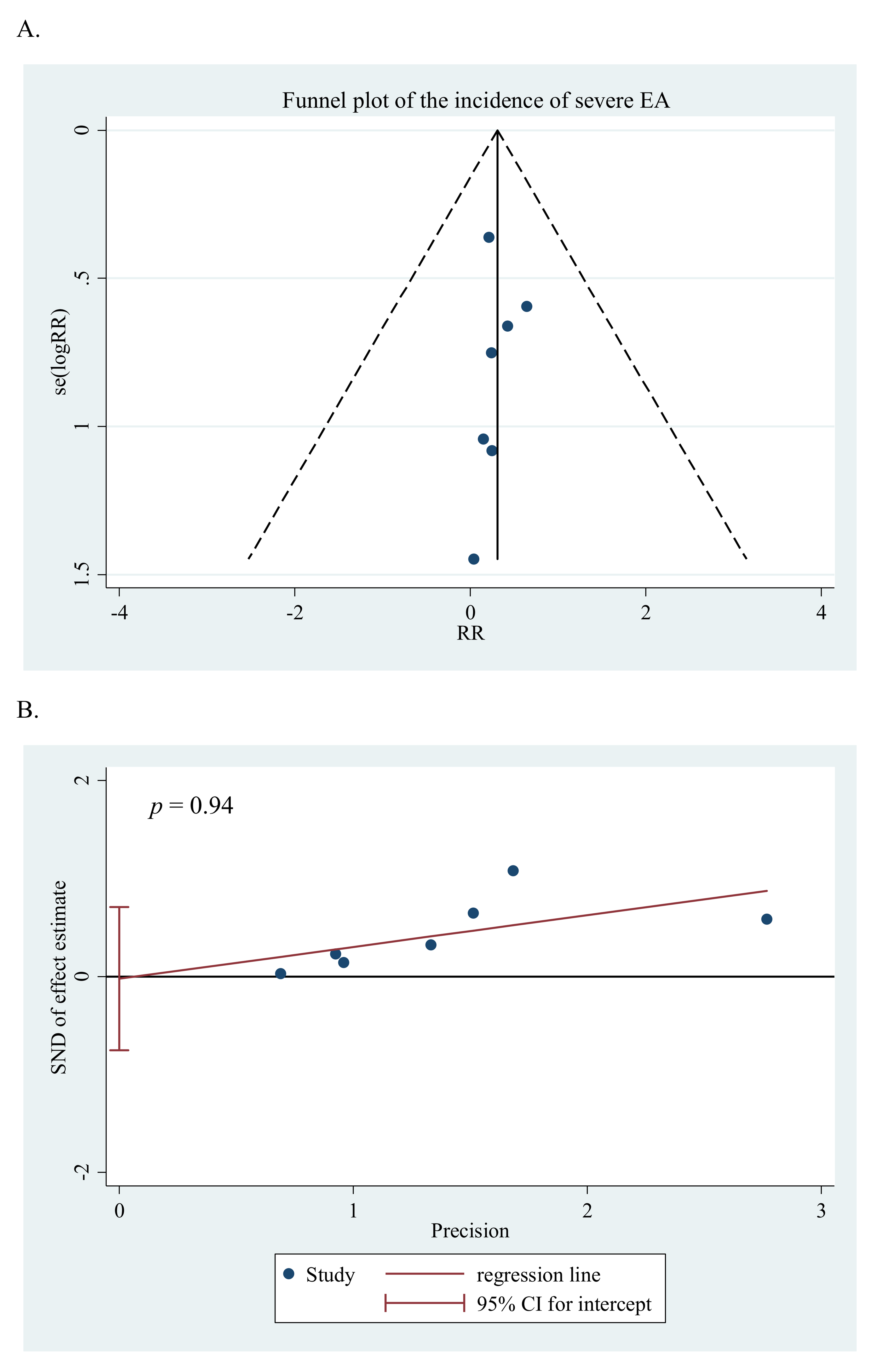

Supplement: S7 Fig — (A) Funnel plots and (B) Egger’s test results for severe EA incidence. The pseudo 95% CIs computed in the analyses were used to obtain the funnel plots and Egger’s test results. These CIs corresponded to the expected 95% CI for a given SE. (TIF) [file pone.0240553.s009.tif]

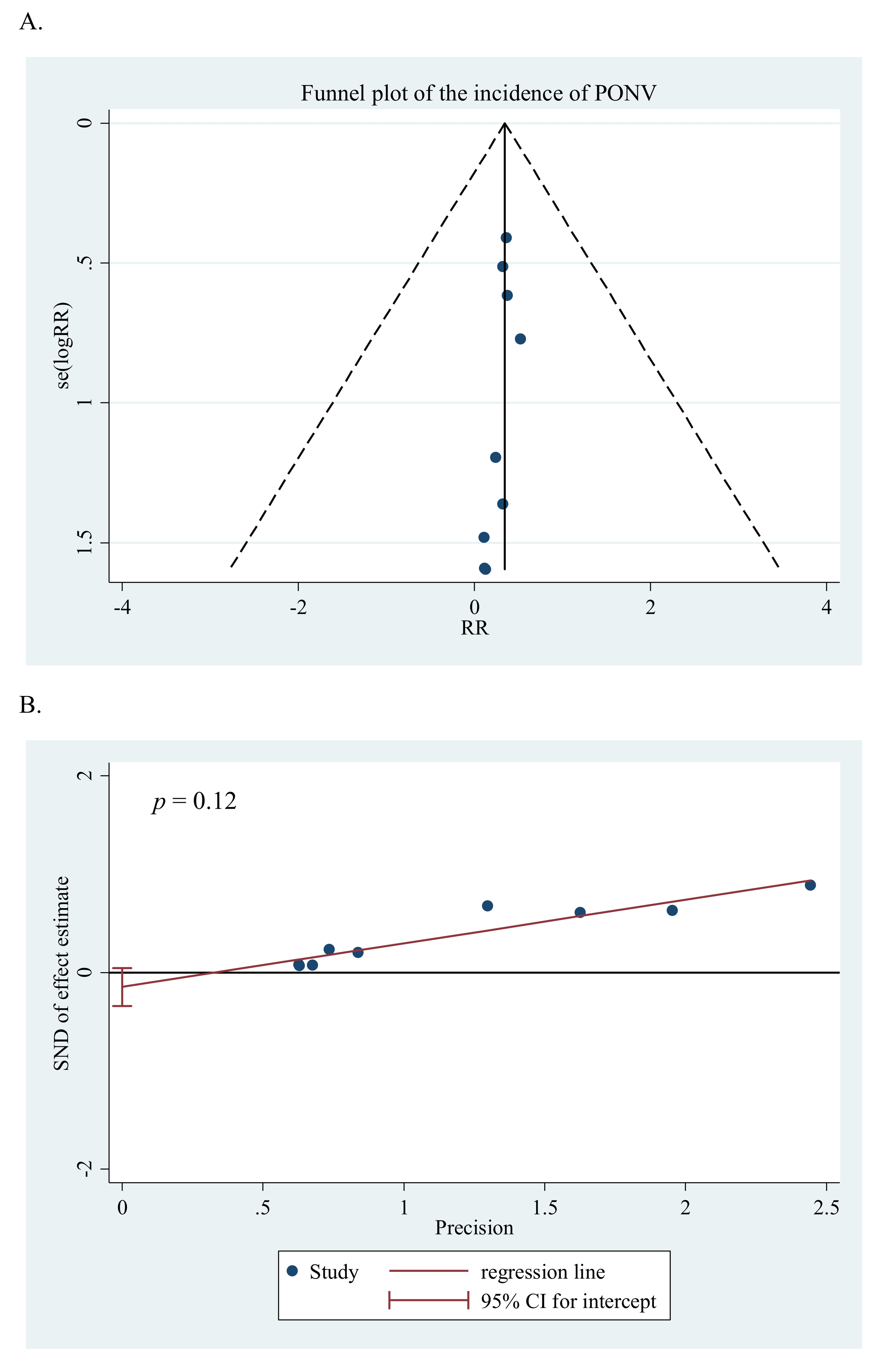

Supplement: S8 Fig — (A) Funnel plots and (B) Egger’s test results for PONV incidence. The pseudo 95% CIs computed in the analyses were used to obtain the funnel plot and Egger’s test results. These CIs corresponded to the expected 95% CIs for a given SE. (TIF) [file pone.0240553.s010.tif]

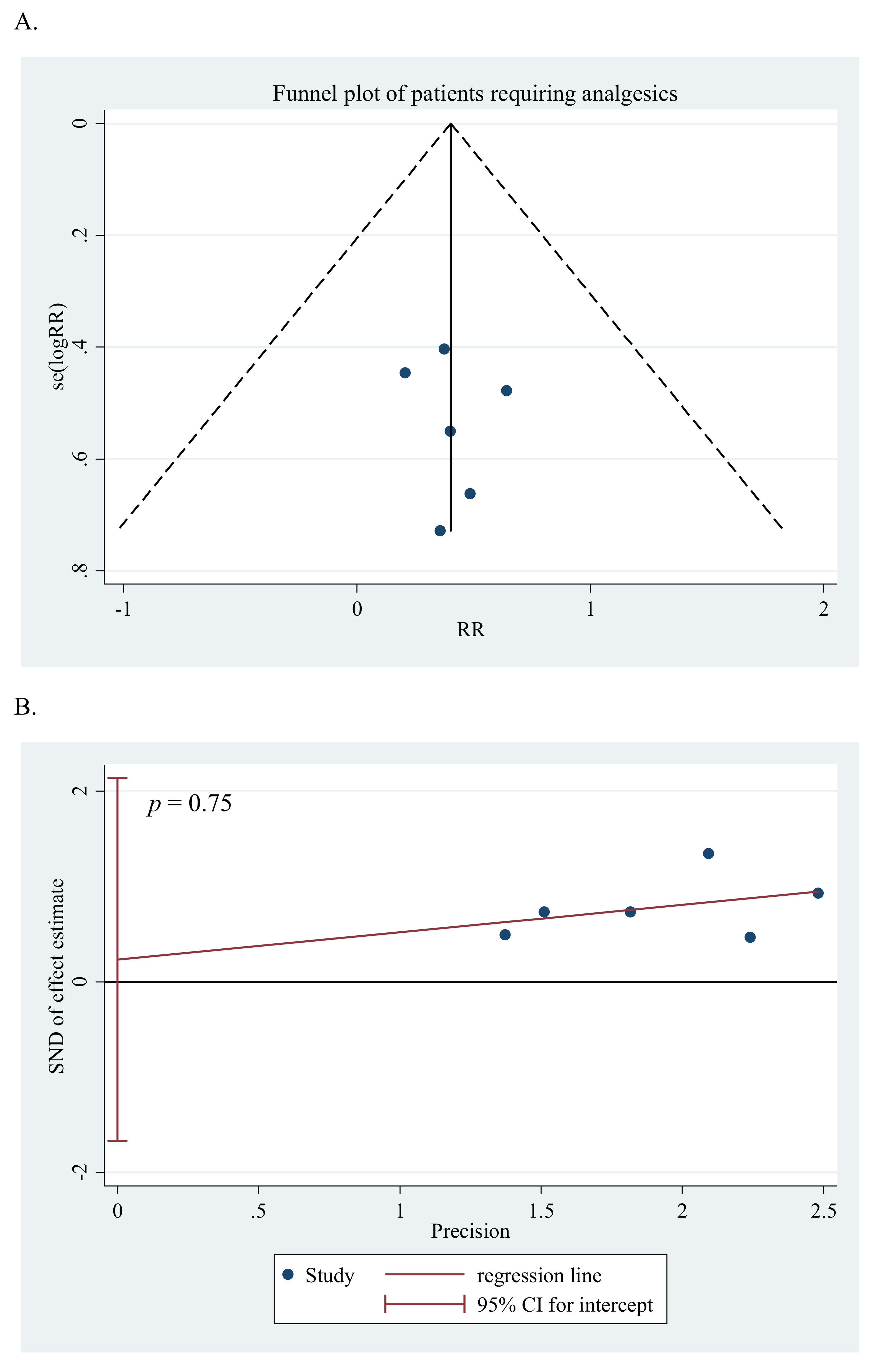

Supplement: S9 Fig — (A) Funnel plots and (B) Egger’s test results for the number of patients requiring rescue analgesia. The pseudo 95% CIs computed in the analyses were used to obtain the funnel plots and Egger’s test results. These CIs correspond to the expected 95% CIs for a given SE. (TIF) [file pone.0240553.s011.tif]

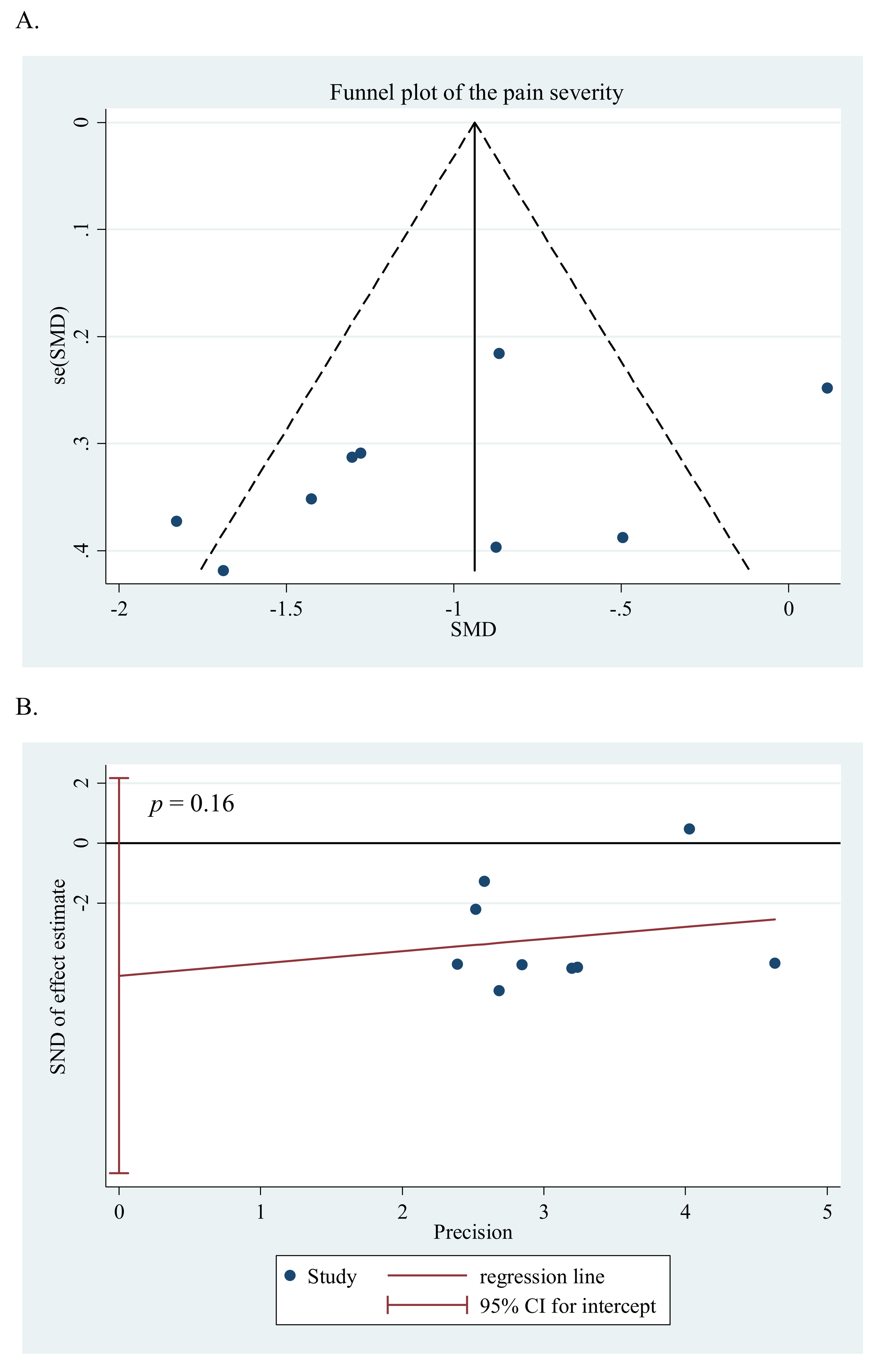

Supplement: S10 Fig — (A) Funnel plots and (B) Egger’s test results for pain scores. The pseudo 95% CIs computed in the analyses were used to obtain the funnel plots and Egger’s test results. These CIs corresponded to the expected 95% CIs for a given SE. (TIF) [file pone.0240553.s012.tif]

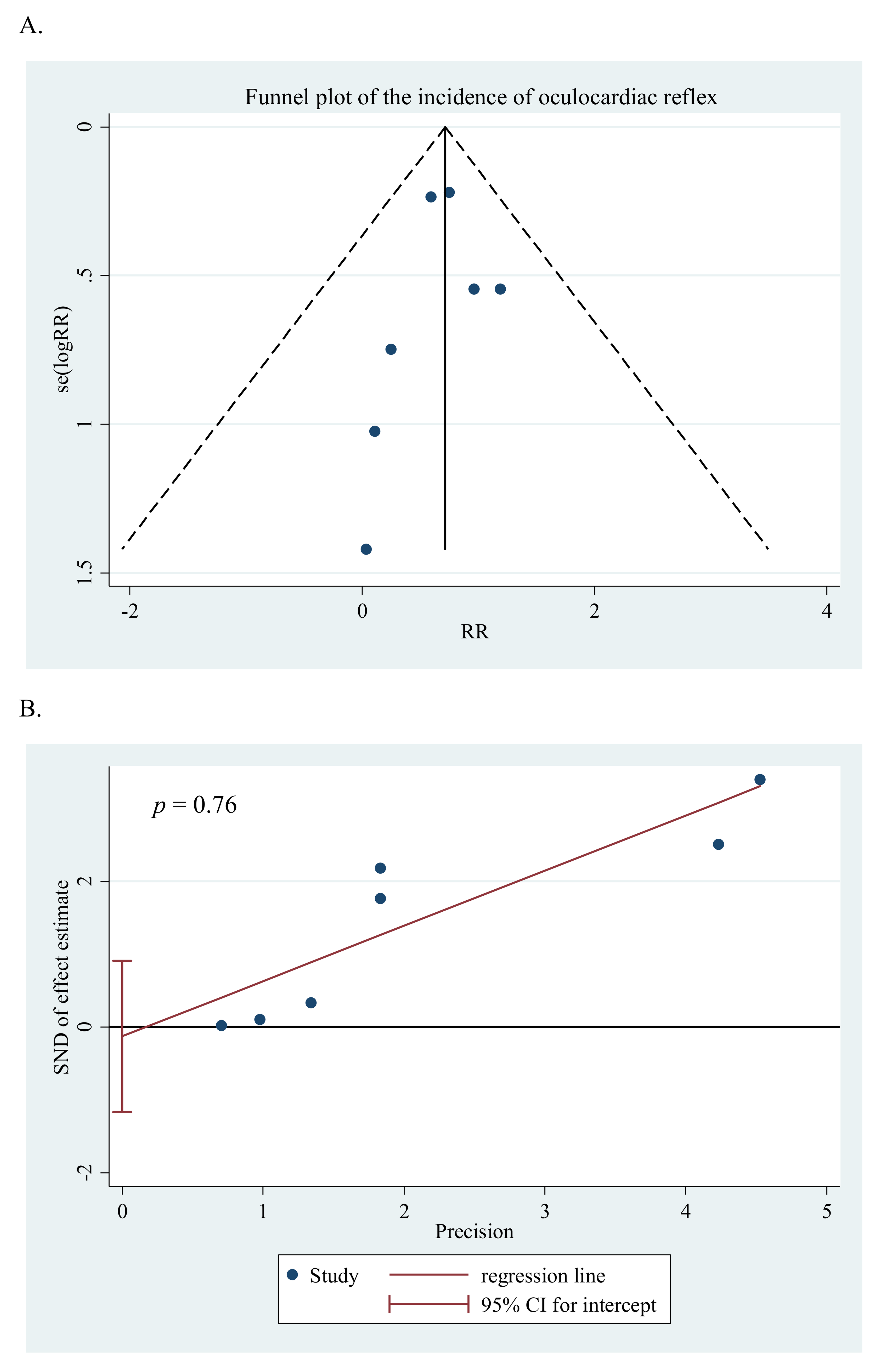

Supplement: S11 Fig — (A) Funnel plots and (B) Egger’s test results for OCR incidence. The pseudo 95% CIs computed in the analyses were used to obtain the funnel plot and Egger’s test results. These CIs corresponded to the expected 95% CI for a given SE. (TIF) [file pone.0240553.s013.tif]

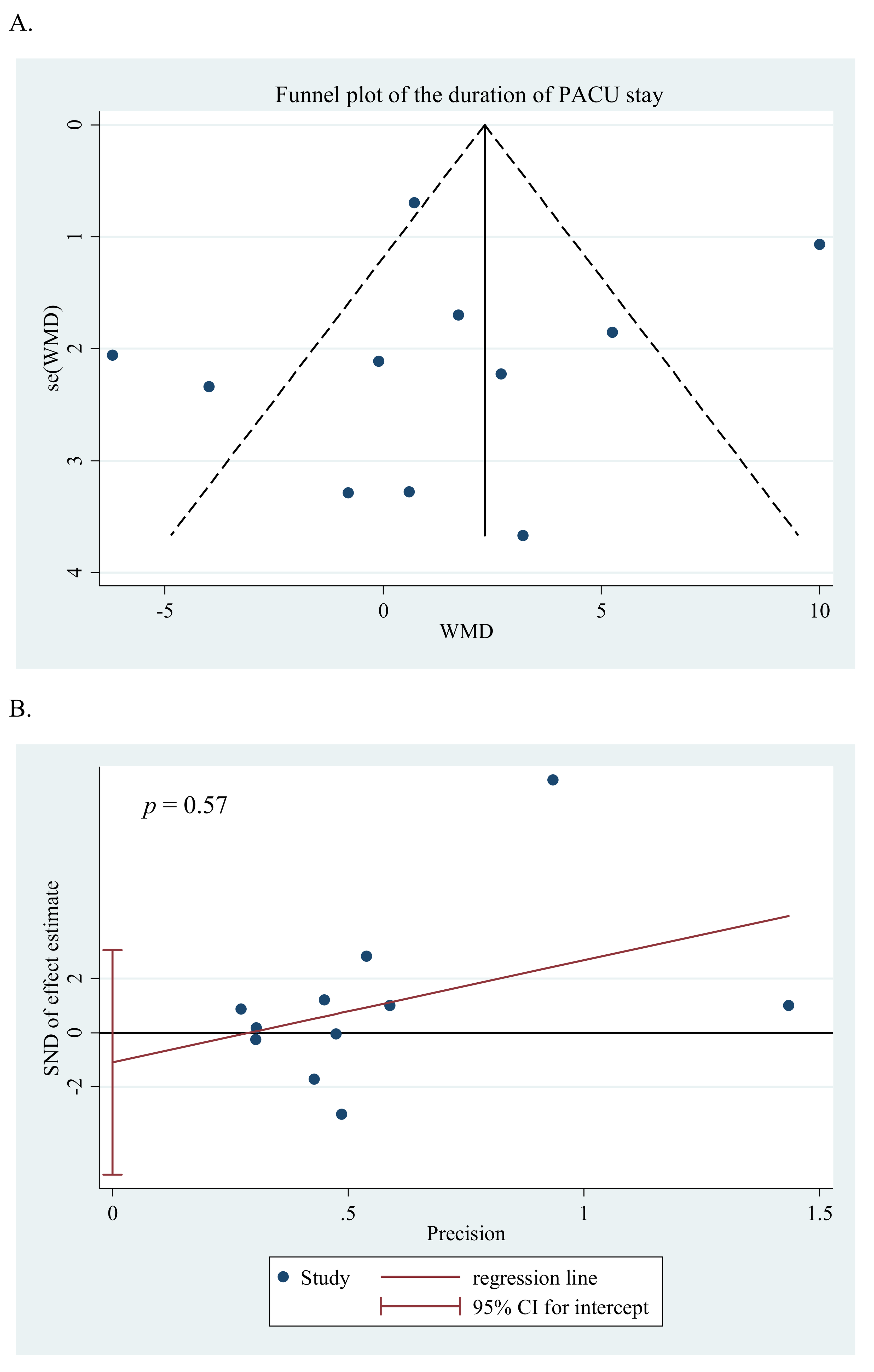

Supplement: S12 Fig — (A) Funnel plots and (B) Egger’s test results for PACU stay duration. The pseudo 95% CIs computed in the analyses were used to obtain the funnel plots and Egger’s test results. These CIs corresponded to the expected 95% CIs for a given SE. (TIF) [file pone.0240553.s014.tif]

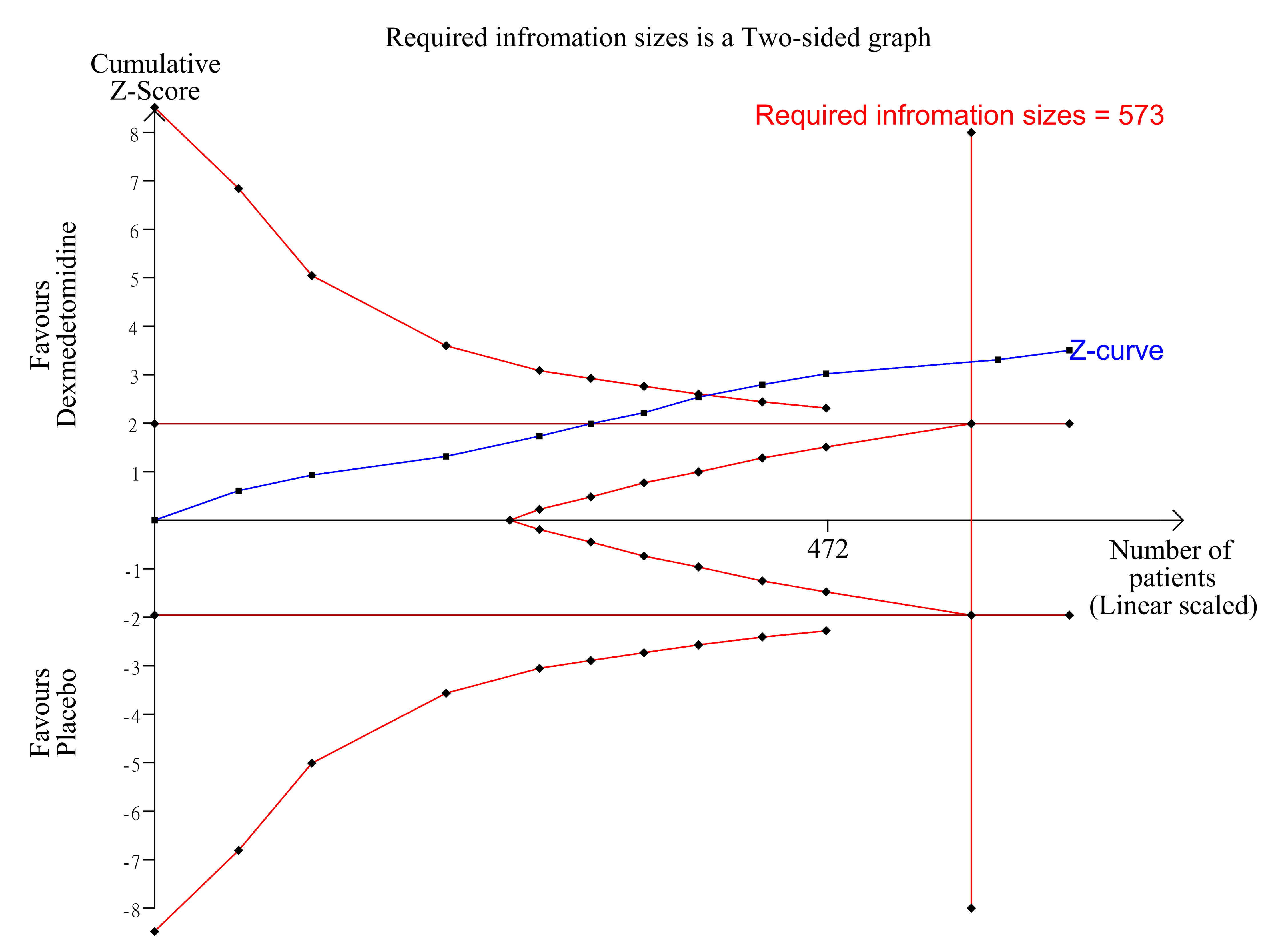

Supplement: S13 Fig — The risk of a type I error was maintained at 5% with 90% power. The variance was calculated from data obtained from the trials included in this meta-analysis. A clinically meaningful intervention effect for EA incidence was set to a 50% relative risk reduction based on an assumption of a 47% proportion of the control group. The result showed solid evidence indicating dexmedetomidine had a lower EA incidence compared to placebo. (TIF) [file pone.0240553.s015.tif]

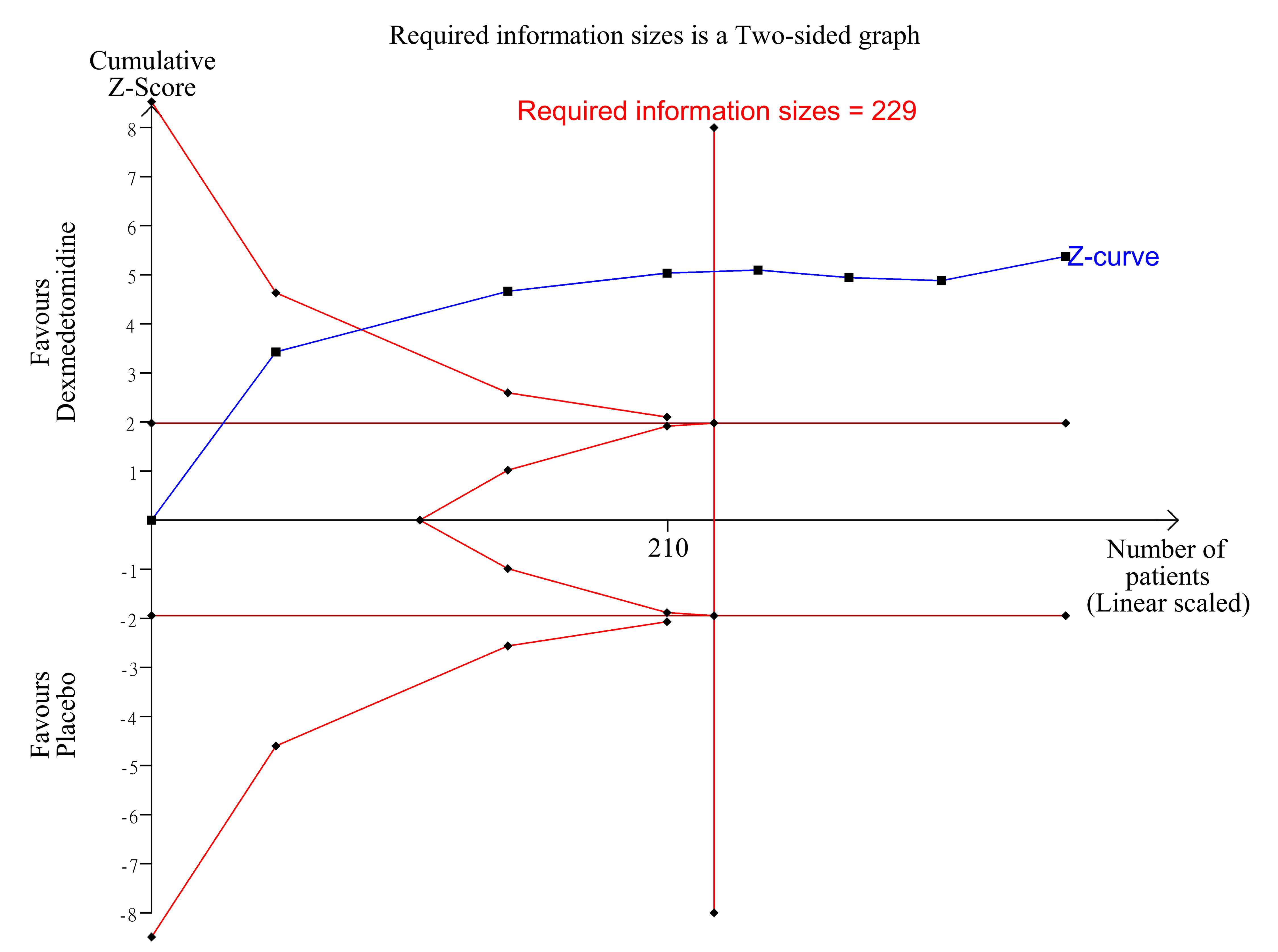

Supplement: S14 Fig — The risk of a type I error was maintained at 5% with 90% power. The variance was calculated from data obtained from the trials included in this meta-analysis. A clinically meaningful intervention effect for severe EA incidence was set to a 50% relative risk reduction based on an assumption of a 39% proportion of the control group. The result showed solid evidence indicating dexmedetomidine had a lower severe EA incidence compared to placebo. (TIF) [file pone.0240553.s016.tif]

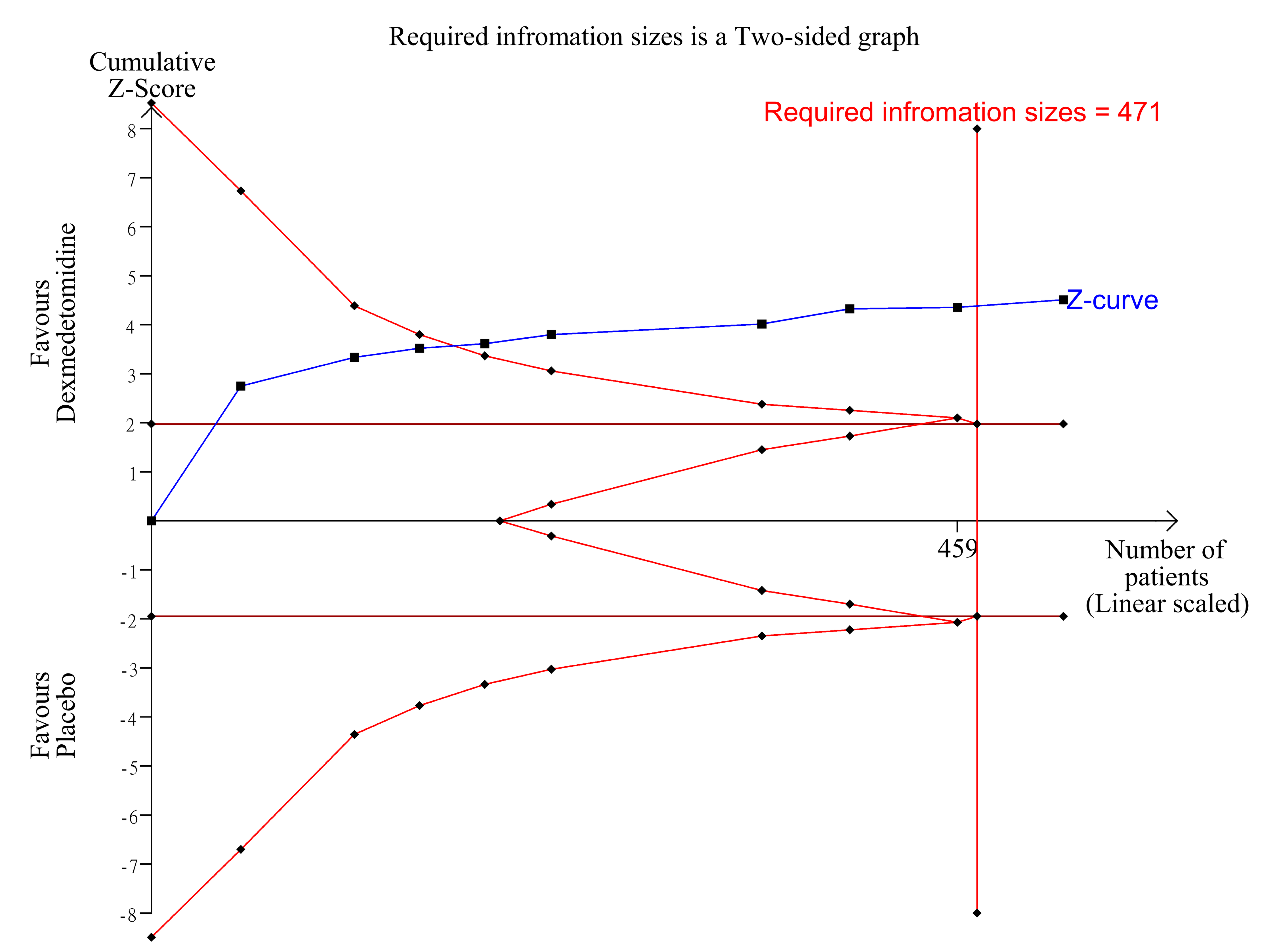

Supplement: S15 Fig — The risk of a type I error was maintained at 5% with 90% power. The variance was calculated from data obtained from the trials included in this meta-analysis. A clinically meaningful intervention effect for PONV incidence was set to a 50% relative risk reduction based on an assumption of a 22.3% proportion of the control group. The result showed solid evidence indicating dexmedetomidine had a lower PONV incidence compared to placebo. (TIF) [file pone.0240553.s017.tif]

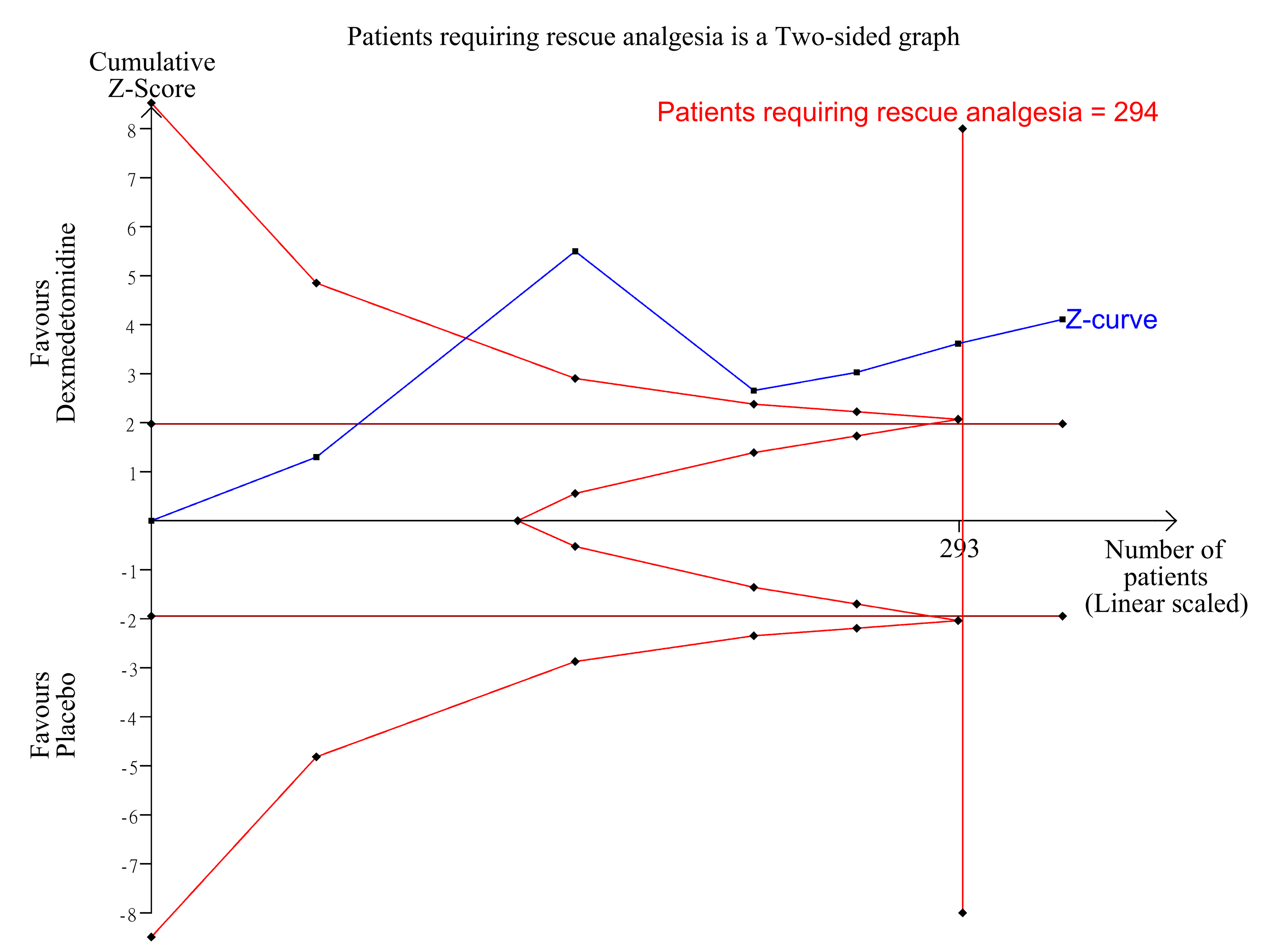

Supplement: S16 Fig — The risk of a type I error was maintained at 5% with 90% power. The variance was calculated from data obtained from the trials included in this meta-analysis. A clinically meaningful intervention effect for patients requiring rescue analgesia was set to a 50% relative risk reduction based on an assumption of a 42% proportion of the control group. The result showed solid evidence indicating dexmedetomidine had a lower proportion of patients requiring rescue analgesia compared to placebo. (TIF) [file pone.0240553.s018.tif]

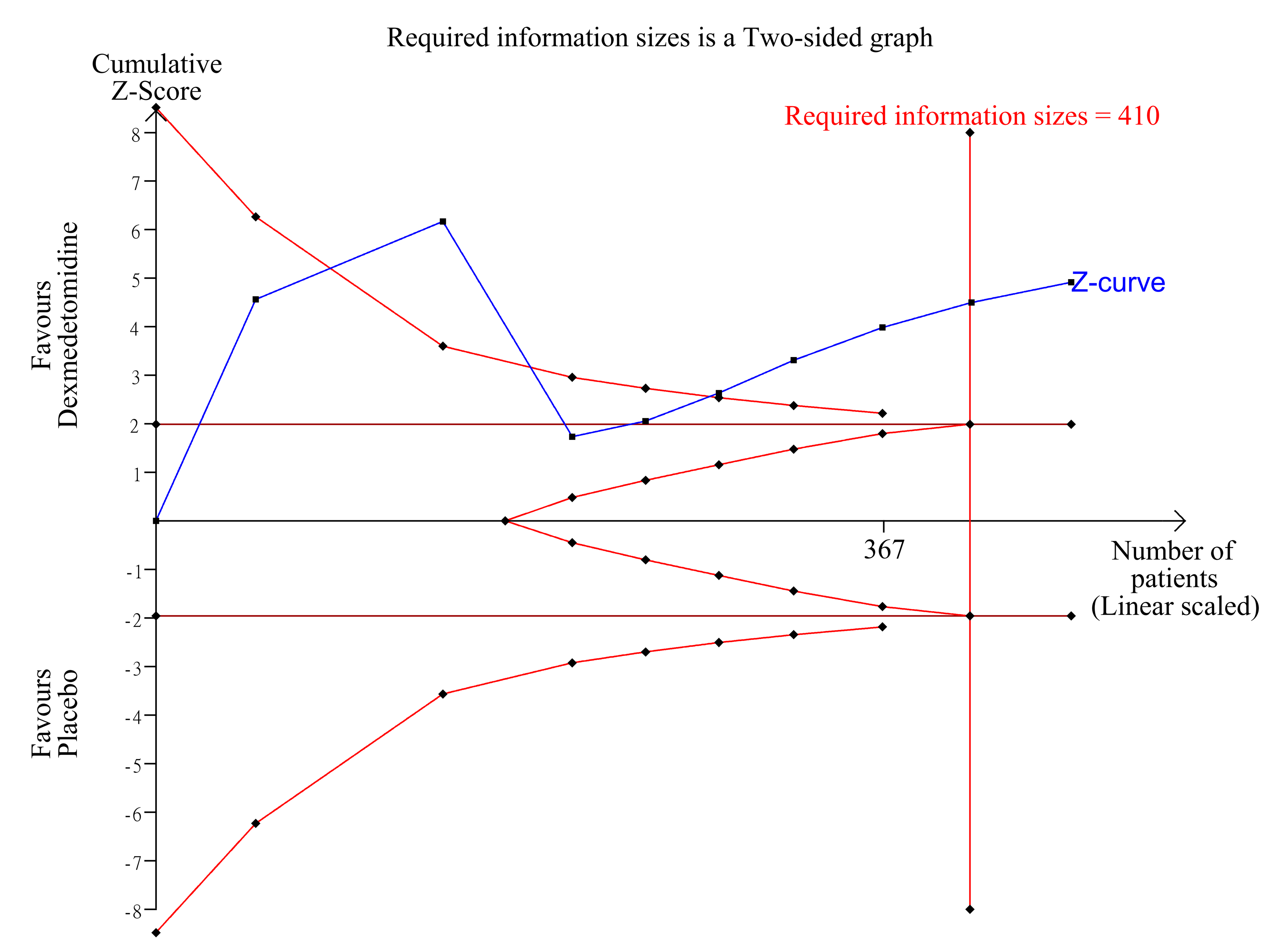

Supplement: S17 Fig — The risk of a type 1 error was maintained at 5% with a power of 90%. The variance was calculated from the data obtained from the included trials. A clinically significant anticipated mean difference in the postoperative pain scores was set to 1.73 based on the pooled result of our meta-analysis. The result showed solid evidence indicating dexmedetomidine had fewer postoperative pain scores compared to placebo. (TIF) [file pone.0240553.s019.tif]

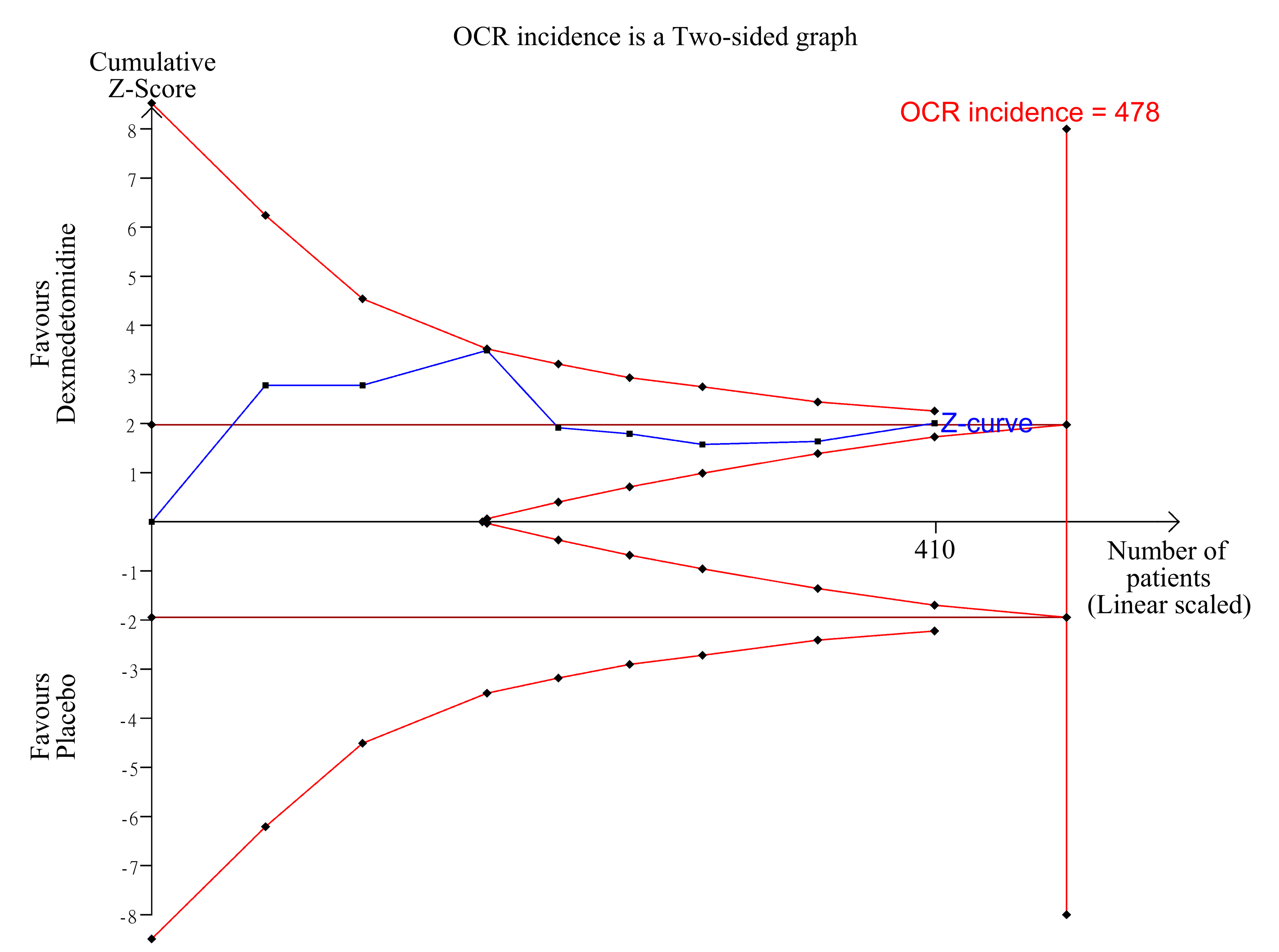

Supplement: S18 Fig — The risk of a type I error was maintained at 5% with 90% power. The variance was calculated from data obtained from the trials included in this meta-analysis. A clinically meaningful intervention effect for OCR incidence was set to a 50% relative risk reduction based on an assumption of a 42.5% proportion of the control group. The result implied that more study needs to be conducted before the effect of dexmedetomidine on the reduction of OCR incidence can be definitively determined. (TIF) [file pone.0240553.s020.tif]

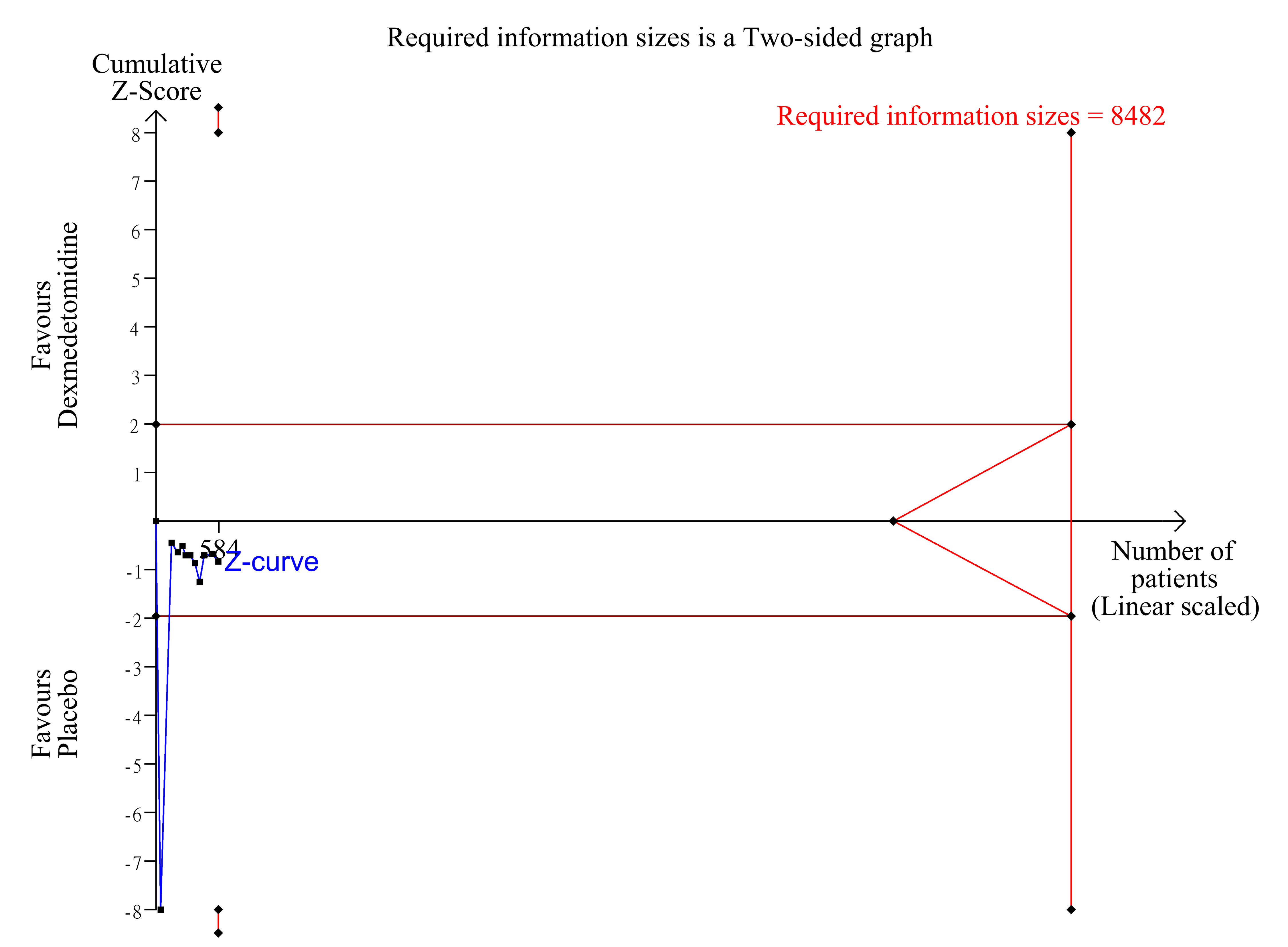

Supplement: S19 Fig — The risk of a type 1 error was maintained at 5% with a power of 90%. The variance was calculated from the data obtained from the included trials. A clinically significant anticipated mean difference in expulsion times was set to 1.33 hours based on the pooled result of our meta-analysis. The result was inconclusive for PACU stay duration which did not differ between the dexmedetomidine and placebo groups. (TIF) [file pone.0240553.s021.tif]
